# Correction: Lu et al. Real-Time 3D Object Detection on Crowded Pedestrians. *Sensors* 2023, *23*, 8725

**DOI:** 10.3390/s25072212

**Published:** 2025-04-01

**Authors:** Bin Lu, Qing Li, Yanju Liang

**Affiliations:** 1 Institute of Microelectronics of the Chinese Academy of Sciences, Beijing 100029, China; lubin@wiot.tech (B.L.); liangyanju@wiot.tech (Y.L.); 2 University of Chinese Academy of Sciences, Beijing 101408, China; 3 Wuxi IoT Innovation Center Co., Ltd., Wuxi 214028, China

Author wants to add a new affiliation and update typographical errors in an existing affiliation.

In the published publication [1], there was an error regarding the Affiliation 1. Besides, a new Affiliation 2 was added. The correct affiliations appear below:

1. Institute of Microelectronics of the Chinese Academy of Sciences, Beijing 100029, China
2. University of Chinese Academy of Sciences, Beijing 101408, China
3. Wuxi IoT Innovation Center Co., Ltd., Wuxi 214028, China

The authors state that the scientific conclusions are unaffected. This correction was approved by the Academic Editor. The original publication has also been updated.

## References

[1] Lu B., Li Q., Liang Y. (2023). Real-Time 3D Object Detection on Crowded Pedestrians. Sensors.

